# *RASSF1A* Promoter Hypermethylation Is a Strong Biomarker of Poor Survival in Patients with Salivary Adenoid Cystic Carcinoma in a Chinese Population

**DOI:** 10.1371/journal.pone.0110159

**Published:** 2014-10-10

**Authors:** Chun-Ye Zhang, Yang-Xing Zhao, Rong-Hui Xia, Jing Han, Bing-Shun Wang, Zhen Tian, Li-Zhen Wang, Yu-Hua Hu, Jiang Li

**Affiliations:** 1 Department of Oral Pathology, 9th People's Hospital, Shanghai Jiao Tong University, School of Medicine, Shanghai Key Laboratory of Stomatology, Shanghai, P. R. China; 2 State Key Laboratory of Oncogenes and Related Genes, Shanghai Cancer Institute, Renji Hospital, Shanghai Jiao Tong University, School of Medicine, Shanghai, P. R. China; 3 Department of Biostatistics, Shanghai Jiao Tong University, School of Medicine, Shanghai, P. R. China; Queen Mary Hospital, Hong Kong

## Abstract

In addition to the clinicopathological parameters, molecular biomarkers are becoming increasingly important in the prognostic evaluation of cancer patients. This study aimed to determine the molecular alterations in the RAS association domain family protein1A gene (*RASSF1A*) in salivary adenoid cystic carcinoma (ACC) and to evaluate the potential of such alterations as prognostic markers. One hundred and sixty-seven ACC tumor tissues and 50 samples of matched normal salivary gland tissues from the same patients were analyzed for *RASSF1A* promoter methylation status by bisulfite sequencing PCR (BSP) and/or methylation-specific PCR (MSP). Fifty ACC tumor tissues and matched normal salivary gland tissues were analyzed for loss of heterozygosity (LOH) by examining two microsatellite markers (D3S1478, D3S1621) at 3p21. *RASSF1A* gene mutations were detected by direct sequencing of all six exons in 50 tumor and normal tissue specimens. Over-all, *RASSF1A* promoter hypermethylation was detected in 35.3% (59/167) of ACC tissues and was associated with histologically solid tumor pattern (*P* = 0.002) and advanced TNM stage (*P* = 0.014). *RASSF1A* LOH was observed in 18.0% (9/50) of cases, and no somatic mutation of *RASSF1A* was detected in any cases. *RASSF1A* promoter methylation was associated with the poor over-all survival (Log-rank test, *P* <0.001) and disease-free survival (Log-rank test, *P* <0.001) and identified as an independent predicator of over-all patient survival (*P* = 0.009) and disease-free survival (*P* <0.001). It was concluded that *RASSF1A* methylation is involved in the development, differentiation and progression of ACC and is a strong independent biomarker of poor survival in ACC patients in a Chinese population.

## Introduction

Salivary adenoid cystic carcinoma (ACC), the most common malignant tumor of salivary glands in our database [1], [2], has certain unique characteristics, such as slow but aggressive growth, nerve and blood invasion, distant metastasis to the lungs at early or late stages and very poor long-term survival [3], [4], [5]. These features make it one of the most characteristic malignant tumors arising from salivary glands. Solid histological pattern and later TNM stage have been confirmed as valid factors for predicting poor prognosis in patients with salivary ACC in our data [6] and other reports [7], [8]. We hypothesized that the clinicopathological parameters of tumor development are accompanied by alterations at the molecular level. Thus, the aim of this study was to understand the molecular basis of ACC tumor development and thereby expand the prospects for developing effective diagnostic and prognostic markers.

Over the past decade, molecular studies have contributed significantly to the identification of genetic and epigenetic changes associated with ACC. In our previous study of 60 ACC tumor tissues [9], we identified the frequent occurrence of promoter methylation of *E-cadherin*, *p16INK4a*, RAS association domain family protein 1A (*RASSF1A*), and death association protein kinase (*DAPK*) genes. However, *RASSF1A* promoter methylation was detected with lower frequency in ACC than in some other tumors [10], [11], [12]. Furthermore, its significant association with tumor histological grade and TNM stage [9], [13] implicated *RASSF1A* as a critical gene in the development of salivary ACC.

*RASSF1A* is a putative tumor suppressor gene located on 3p21 and functions in cell cycle control, microtubule stabilization, cellular adhesion, motility and apoptosis [14]. Promoter methylation, which is an epigenetic change, is the predominant mechanism of *RASSF1A* gene inactivation, and has been recognized in many human solid tumors, including non-small cell lung carcinoma (NSCLC) [15], [16], [17], breast carcinoma [10], [18], [19] and colorectal carcinoma [20], [21]; thus, *RASSF1A* promoter methylation may be a prognostic indicator in such tumors. Loss of heterozygosity (LOH), which is among the genetic changes in regions of the *RASSF1A* gene, has been detected in cervical squamous cell carcinoma and adenocarcinoma [22], [23] and gallbladder carcinoma [24]. *RASSF1A* mutation, another genetic abnormality, has also been found in primary nasopharyngeal carcinoma though no obvious mutational hot-spots were observed [25]. These reports demonstrated that various mechanisms are involved in *RASSF1A* gene inactivation, and that abnormalities in the *RASSF1A* gene may be a crucial event in the development and progression of some types of malignant tumors. To date, there are no reports describing LOH or *RASSF1A* mutations in ACC tumors. Furthermore, the association between molecular changes in the *RASSF1A* gene and tumor survival remains to be clarified. This study, conducted in 167 Chinese patients with salivary ACC, aimed to determine the molecular alterations of *RASSF1A* in salivary ACC and to evaluate the potential of such alterations as prognostic markers. We detected the promoter methylation status, LOH and *RASSF1A* gene mutations in ACC tumors and further elucidated the association of the *RASSF1A* abnormalities with patient outcome.

## Results

### Clinical data

Of the 167 patients with salivary ACC included in this study, 78 were women and 89 were men, and the medium age was 52.5 years (range 27–83). Eighty-one percent (135/167) of the cases exhibited cribriform and tubular patterns, and 19% (32/167) exhibited the solid pattern. Twenty-two of 167 cases (13%) were classified as TNM stage I, 74 cases (44%) as stage II, 53 cases (32%) as stage III and 18 cases (11%) as stage IV (Table S2 in File S1).

### Promoter methylation, LOH and mutation of RASSF1A and correlations between these indexes

**Figure 1:**
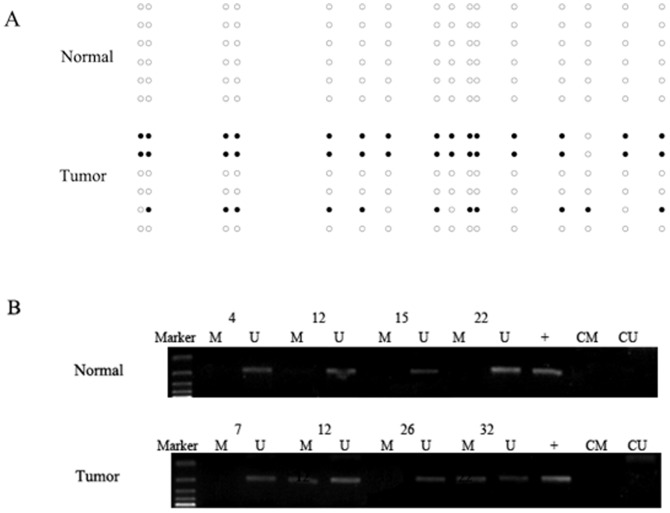
*RASSF1A* promoter methylation analyses in salivary adenoid cystic carcinoma and normal salivary gland tissues. A. *RASSF1A* promoter methylation in case 4 was detected by bisulfite sequencing. Each circle indicates a CpG dinucleotide and each row indicates the sequencing result of one clone. Black and white dots indicate methylation and unmethylation of a CpG dinucleotide, respectively. There was no *RASSF1A* promoter methylation in normal tissue and partial promoter methylation in tumor tissue in case 4. B. MSP analysis of the promoter methylation status of *RASSF1A* in normal and tumor tissues. M indicates DNA amplified with methylation-specific primers; U, DNA amplified with unmethylation-specific primers; +, positive control; CM, blank control for methylation; CU, blank control for unmethylation.

Among 15 paired ACC tumor and normal tissues, methylation of the *RASSF1A* gene promoter was detected in four cases (27%) (cases 4, 5, 9 and 12) by BSP. By MSP, methylation of the *RASSF1A* gene promoter was detected in 59 of 167 (35%) tumor tissues but not in salivary gland tissues (Fig. 1). The results of BSP and MSP were consistent

**Figure 2:**
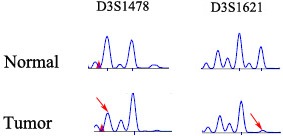
Representative results of detection of LOH in salivary adenoid cystic carcinoma. The markers D3S1478, D3S1621 flanking 3p21 regions were analyzed. Arrows indicate alleles that showed LOH in the tumor samples.

LOH was detected in 9 of 50 (18%) tumors in the *RASSF1A* regions (Fig. 2).

In the 50 cases in which both methylation and LOH were explored, methylation of the *RASSF1A* gene promoter was detected in 17 cases, with concurrent LOH of *RASSF1A* detected in six cases. Three cases with LOH of *RASSF1A* without methylation of the *RASSF1A* gene promoter were found. No association was identified between LOH and methylation of the *RASSF1A* gene promoter (*P* = 0.467).

**Figure 3:**
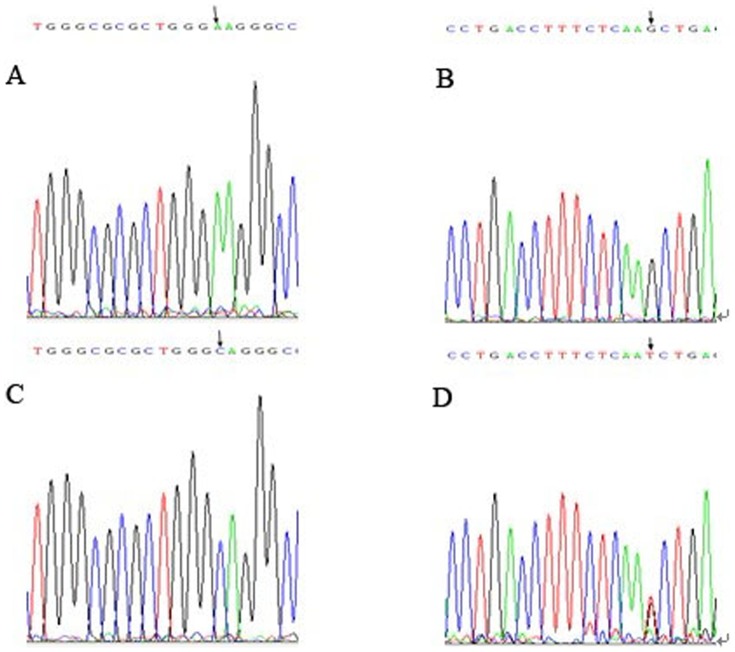
Mutational analyses of *RASSF1A* in salivary adenoid cystic carcinoma. A and B, Wild-type sequences. C and D, Polymorphisms at codon 21 (AAG to CAG) and codon 133 (GCT to TCT) were noted (arrow).

In 27 of 50 (54%) ACC tumors and matched salivary gland tissues and also in 13 of 20 (65%) peripheral blood cells from healthy donors, an identical transversion from A to C was found in exon 1 of the *RASSF1A* gene. This corresponds to a sequence change from AAG to CAG at codon 21 (Fig. 3), resulting in a lysine to glutamine amino acid substitution. Similarly, a sequence change from GCT to TCT at codon 133 (Fig. 3), resulting in an alanine to serine substitution, was identified in exon 3 of the *RASSF1A* gene in 5 of 50 (10%) ACC tumors and matched salivary gland tissues, and in 2 of 20 (10%) peripheral blood cells of healthy donors. No nucleotide alterations were found in exons 2, 4, 5 and 6 of the *RASSF1A* gene, either in salivary ACC patients or in healthy volunteers. No statistical differences in the nucleotide alterations in exon 1 and exon 3 between salivary ACC patients and healthy individuals (*P* = 0.560, *P* = 0.680, respectively) indicated that these changes were germline polymorphisms rather than somatic mutations. No somatic mutations were detected in any tumor or normal specimen.

### The association between the promoter methylation, LOH of RASSF1A and clinicopathological parameters

According to the results of MSP, *RASSF1A* promoter methylation was correlated with the solid histological pattern (*P* = 0.002) and advanced clinical stage (*P* = 0.014) (Table 1).

**Table 1:** Association between *RASSF1A* promoter hypermethylation status and clinicopathologic characteristics.

Clinicopathologic characteristics		Methylation		*P*
		Positive	Negative	
Age	<60 y	42	75	0.861
	≥60 y	17	33	
Sex	Male	35	54	0.261
	Female	24	54	
Location	Minor	38	76	0.488
	Major	21	32	
Histological pattern	Cribriform and tubular	40	95	**0.002**
	Solid	19	13	
Stage	I and II	26	70	**0.014**
	III and IV	33	38	
Neural/vascular invasion	Yes	54	94	0.453
	No	5	14	

No correlation was identified between LOH of *RASSF1A* and the age (*P* = 0.442), and sex (*P* = 0.479) of patients, and the location (*P* = 0.127), histological pattern (*P* = 0.595), TNM stage (*P* = 0.281) and perineural/vascular invasion (*P* = 1.000) of the tumors.

### Survival analysis

**Figure 4:**
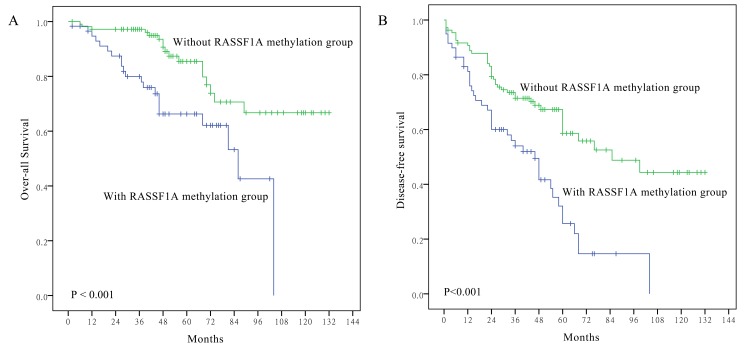
Kaplan–Meier survival curves according to *RASSF1A* promoter methylation status. Patients with *RASSF1A* promoter methylation showed poorer over-all survival (A) (*P* <0.001) and disease-free survival (B) than those without (*P* <0.001).

**Figure 5:**
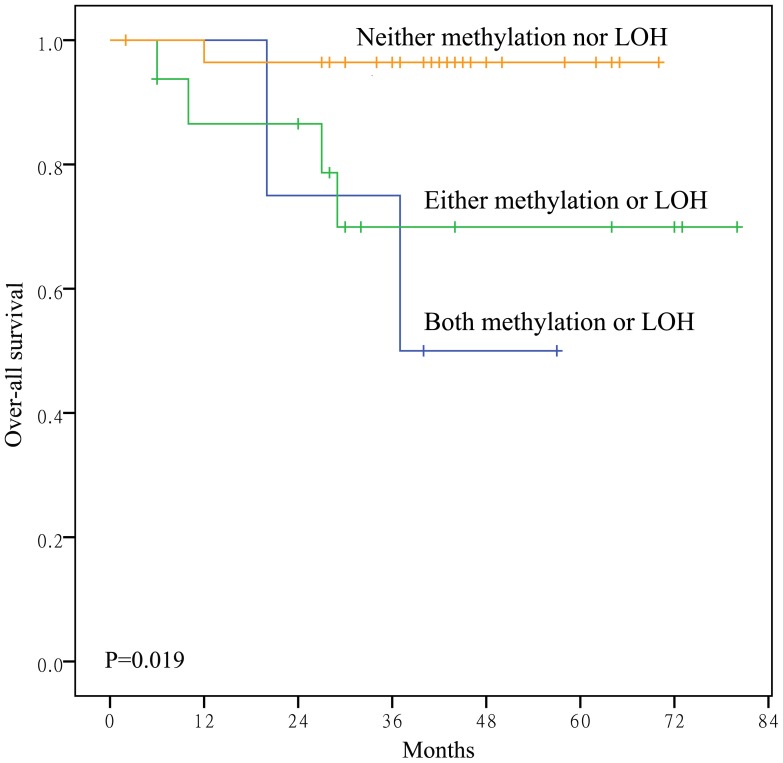
Kaplan–Meier survival curves according to promoter methylation status and LOH of *RASSF1A*. Patients with concurrent promoter methylation and LOH of *RASSF1A* showed the poorest over-all survival (*P* = 0.019) among the three groups.

Survival curves were generated for all 167 salivary ACC cases. Methylation of the *RASSF1A* gene promoter was significantly associated with over-all survival (Log-rank test, *P* <0.001) and disease-free survival (Log-rank test, *P* <0.001) (Fig. 4). Based on *RASSF1A* methylation and LOH status, patients were divided into three groups: neither methylation nor LOH, either methylation or LOH, and both methylation and LOH. Statistically significant differences in over-all patient survival were identified among these three groups (Fig. 5) when analyzed with the Cox proportional hazards model. In univariate analyses, older patients (*P <*0.001), solid histological pattern (*P <*0.001), advanced TNM stage (*P* = 0.045) and *RASSF1A* promoter methylation (*P* = 0.001) were associated with poorer over-all survival (Table 2). Furthermore, older patients (*P* = 0.002), solid histological pattern (*P* = 0.004), advanced TNM stage (*P* = 0.045) and *RASSF1A* promoter methylation (*P <*0.001) were associated with worse disease-free survival (Table 3).

**Table 2:** Summary of Cox model estimates for over-all survival in 167 patients with ACC.

Variables	Hazard ratio	95% Hazard ratio confidence limits	*P*
**Univariate**			
Age <60 vs. ≥60	0.311	0.165–0.583	<0.001
Sex male vs. female	1.529	0.803–2.913	0.196
Location major vs. minor	1.450	0.706–2.977	0.312
Histological type c/t vs. s	0.297	0.156–0.566	<0.001
TNM Stage I/II vs. III/IV	0.522	0.277–0.985	0.045
Margin negative vs. positive	0.857	0.440–1.671	0.651
Neural/vascular invasion positive vs. negative	1.165	0.453–2.996	0.751
Radiotherapy and/or chemotherapy yes vs. no	0.963	0.437–2.118	0.924
* RASSF1A* methylation positive vs. negative	3.051	1.608–5.788	0.001
**Multivariate**			
Age <60 vs. ≥60	0.315	0.166–0.596	<0.001
Histological type c/t vs. s	0.350	0.178–0.688	0.002
TNM Stage I/II vs. III/IV	0.822	0.417–1.618	0.570
* RASSF1A* methylation positive vs. negative	3.146	1.618–6.119	0.001

**Table 3:** Summary of Cox model estimates for disease-free survival in 167 patients with ACC.

Variables	Hazard ratio	95% Hazard ratio confidence limits	*P*
**Univarate**			
Age <60 vs. ≥60	2.031	1.298–3.180	0.002
Sex male vs. female	0.727	0.467–1.131	0.158
Location major vs. minor	0.725	0.446–1.179	0.195
Histological type c/t vs. s	2.067	1.264–3.378	0.004
TNM Stage I/II vs. III/IV	1.562	1.009–2.417	0.045
Margin negative vs. positive	1.618	1.030–2.541	1.618
Neural/vascular invasion positive vs. negative	0.550	0.252–1.201	0.134
Radiotherapy and/or chemotherapy yes vs. no	1.079	0.640–1.816	0.776
* RASSF1A* methylation positive vs. negative	0.415	0.267–0.644	<0.001
**Multivariate**			
Age <60 vs. ≥60	1.892	1.206–2.907	0.006
Histological type c/t vs. s	1.600	0.961–2.665	0.071
TNM Stage I/II vs. III/IV	1.136	0.715–1.807	0.589
Margin negative vs. positive	1.507	0.949–2.392	0.082
* RASSF1A* methylation positive vs. negative	0.434	0.275–0.684	<0.001

To determine whether the association between *RASSF1A* promoter methylation and survival was independent of other parameters, multivariate analysis was performed in which patient age, histological type, TNM stage and *RASSF1A* promoter methylation were included as co-factors. Older patients (*P* <0.001), solid histological pattern (*P* = 0.002) and *RASSF1A* promoter methylation (*P* = 0.001) were independently associated with over-all survival (Table 2). Older patients (*P* = 0.006) and *RASSF1A* promoter methylation (*P* <0.001) were also independently associated with disease-free survival (Table 3).

## Discussion

In this study, solid tumor pattern, later TNM stage and greater age were identified as predicators of poor outcome in salivary ACC patients, which is consistent with our previous report [6]. However, in this study, we investigated whether the genetic and epigenetic alterations of *RASSF1A* could be used in addition to the clinicopathological features as prognostic biomarkers in salivary ACC. One of the characteristics of our study was the combination of the BSP and MSP techniques to determine the methylation status of *RASSF1A*. BSP, which has served as the “gold standard” for determining DNA methylation, has the advantage that it allows the extent of methylation to be determined by detecting each CpG site. However, this approach is quite costly and time-consuming if a large number of cases and clones are sequenced [26]. MSP is a rapid and sensitive technique for assessing the methylation status in CpG islands and can be used for lower quantity and/or quality DNA samples [27], [28]. However, due to the experimental design and variables, MSP is associated with a high false positive and negative detection rate [29]. Therefore, in this study, we employed both BSP and MSP to evaluate DNA methylation; the results of the two techniques were consistent, indicating the reliability of our data.

Our previous report describing the analysis of 60 salivary ACC from our 1999–2002 cohort showed that *RASSF1A* gene promoter methylation was correlated with the grade and the TNM stage of salivary ACC [9]. In the current study, we used a larger sample group (167 cases) from our 2003–2008 data to further confirm and investigate the role of *RASSF1A* in ACC tumors. *RASSF1A* promoter methylation was detected in 35% of salivary ACC tumors in this study by BSP and MSP, which is consistent with our earlier findings [9]. Our study indicated that the methylation ratio of *RASSF1A* in ACC was lower than in many other tumors, such as the primary lung cancer (88%) [30], breast carcinoma (65%–95%) [31], [32], [33] and colorectal carcinoma(52%) [21]. However, the fact that gene methylation was associated with solid tumor pattern and advanced ACC stage suggested that the *RASSF1A* gene may be particularly important in cell differentiation and tumor progression in ACC. Similar conclusions have been reported in bladder cancer [34] and Wilms' tumor [35].

According to the two-hit theory, biallelic abnormalities of tumor suppressor genes can result in complete inactivation of the gene and promote tumorigenesis. The protein encoded by *RASSF1A* modulates a broad range of cellular functions. Although *RASSF1A* can be inactivated by LOH or point mutation, the most common contributor to loss or reduction of *RASSF1A* function is the transcriptional silencing of the gene by inappropriate promoter methylation [36]. Our results confirmed that promoter methylation was the most common (35%) molecular abnormality of the *RASSF1A* gene in salivary ACC. Additionally, LOH (9/50, 18%) was involved in *RASSF1A* gene inactivation in some ACC tumors. The two-hit phenomenon, which is constituted by *RASSF1A* gene promoter methylation and LOH at 3p21 was detected in six ACC samples (6/50, 12%). The relatively lower chance of the two-hit phenomenon in the *RASSF1A* gene has also been reported by Choi et al. and Yu et al. in cervical cancers [22], [23].

Somatic mutation of the *RASSF1A* gene has rarely been reported in human cancers. Only 9.5% of primary nasopharyngeal carcinomas [37] and 10% of lung tumors [11] carry missense mutations. In this study, no somatic mutations were detected by sequencing all six exons of the *RASSF1A* gene for the 50 matched cancerous and noncancerous tissues. However, a single nucleotide germline polymorphism at codon 133 of exon 3 was detected in five of the tumor tissues. This polymorphism has also been reported in cervical cancer [23], nasopharyngeal cancer [37]. Moreover, in many cases (27/50, 54%) of in this study, a single germline polymorphism was detected at codon 21 of exon 1, which has not been reported in other tumors.

As a characteristic tumor, there are many reports [7], [8] describing the association of the clinicopathological features of salivary ACC and the prognosis of patients. It is equally important to determine characteristic molecular abnormalities that represent prognostic molecular biomarkers in ACC patients. Our study confirmed the role of clinicopathological parameters such as solid tumor pattern, later TNM stage and greater age in predicting the outcome of ACC patients. Our study is the first to report a correlation between *RASSF1A* gene promoter methylation and outcome in salivary ACC patients. In our data, *RASSF1A* promoter methylation was a strong predicator of disease-free survival and over-all survival in patients with salivary ACC, both in univariate and multivariate survival analyses. *RASSF1A* gene promoter methylation has also been reported to confer poorer survival in surgically treated NSCLC [15], breast cancer [19], [33], stage I and II lung cancer [38] and Wilms' tumor [35]. Our findings provide evidence of the potential usefulness of *RASSF1A* promoter methylation as an informative prognostic biomarker in patients with ACC.

Our study also demonstrated the potential of promoter methylation and LOH of *RASSF1A* as molecular markers to predict salivary ACC patient outcome. Survival differences were compared among the 50 cases following allocation to three groups according to *RASSF1A* methylation and LOH status. Patients with concurrent *RASSF1A* methylation and LOH at 3p21 had the poorest prognosis compared to patients with either *RASSF1A* methylation or LOH at 3p21 and those with neither *RASSF1A* methylation nor LOH at 3p21. This suggested that inactivation of both *RASSF1A* alleles had the greatest influence on tumor progression, whereas partial *RASSF1A* gene function is provided by one wild-type allele. However, it should be noted the sample size for detection of LOH of RASSF1A in this study was relatively small; therefore, LOH frequency in ACC may be biased. Further studies are necessary to determine the roles of *RASSF1A* LOH in ACC.

In summary, the present study indicated that DNA promoter methylation is the most common molecular abnormity of the *RASSF1A* gene in salivary ACC. Moreover, *RASSF1A* gene promoter methylation may play a significant role in ACC carcinogenesis progression and may be a reliable prognostic biomarker of poor patient survival. Further studies on the correlation between abnormalities in the *RASSF1A* gene and protein expression as well as interactions with other genes in salivary ACC are therefore warranted.

## Materials and Methods

### Ethics statement

This study was approved by the Human Research Ethics Committee of Shanghai Ninth People's Hospital, Shanghai Jiao Tong University School of Medicine, and written informed consent was obtained from all patients.

### Tissue samples

One hundred and sixty-seven salivary ACC tumors and 50 samples of matched normal salivary gland tissues from the same patients were collected from ACC surgical patients at the Shanghai 9^th^ People's Hospital, Shanghai Jiao Tong University, Shanghai, China, from 2003 to 2008. All samples were fixed in formalin and embedded in paraffin. The tissue sections (4 µm) were stained with hematoxylin and eosin and all cases were reviewed by two pathologists to confirm the diagnosis. For cases with non typical morphology, the diagnosis was based primarily on immunohistochemical staining of CK7 (Gene Tech, USA), CK8 (Gene Tech, USA), c-kit(Gene Tech, USA), S-100(Gene Tech, USA), SMA (Gene Tech, USA), Calp (Gene Tech, USA), MYB(abcam, UK) and fluoresence in situ hybridization (FISH) for MYB using a dual-color MYB break-apart probe (Zytovision, German).

The histological pattern of each tumor was determined according to the histological typing of salivary gland tumors defined by the World Health Organization [39], and the ACC tumors were subclassified with cribriform, tubular and solid patterns. When more than 30% of tumor had a solid component, it was classified as having a solid histological pattern. Tumors were staged in accordance with the AJCC cancer staging criteria based on clinical findings and preoperative imaging [40].

To identify the most frequently occurring methylation sites of the CpG islands located in the *RASSF1A* gene in ACC tumors, 15 tumors and their matched normal tissues were selected at random from the 167 cases and subjected to bisulfite sequencing PCR (BSP). The primer pairs for methylation-specific PCR (MSP) were designed according to the results of bisulfite sequencing. In many cases, the normal tissues were not available; therefore, 167 ACC tumors and 50 matched normal tissues were subjected to MSP analysis.

Tumor tissue and matched normal tissue are required for LOH and mutation analysis; therefore, of the 167 cases, only those 50 cases for which the tumor tissues and matched normal tissues were available were analyzed for DNA methylation, LOH and mutations of the *RASSF1A* gene. DNA extracted from peripheral blood cells of 20 healthy volunteers was used as a normal control for DNA polymorphism evaluation of the germline cells.

### DNA extraction

DNA was extracted from formalin-fixed, paraffin-embedded tissues using the QIAamp DNA FFPE Tissue kit (Qiagen) according to the manufacturer's instructions.

### Methylation analysis by bisulfite sequencing PCR (BSP) and methylation-specific PCR (MSP)

For BSP, genomic DNA was modified by sodium bisulfite treatment and purified using EpiTect Bisulfite Kit (Qiagen) according to the recommendations of the manufacturer. The sequences of the primers and the amplification products, which included 16 CpG islands of the promoter region of *RASSF1A* gene, are listed in Table S1 in File S1. The amplified fragments were subcloned. Six clones of each sample were selected for sequencing. The 16 CpG islands were screened for hot-spot methylation sites.

For MSP, genomic DNA was modified by sodium bisulfite treatment. The detailed sequences of primers are listed in Table S1 in File S1. The PCR amplification was performed in a 12.5 µl reaction volume with HotStar Taq DNA polymerase (Qiagen) according to the manufacturer's instructions. Water was substituted for DNA in the blank control. DNA extracted from normal lymphocytes and treated with *Sss*I methylase (New England Biolabs, Beverly, MA, USA) was used as the positive control. The PCR products were subjected to electrophoresis on 2% agarose gels, stained with ethidium bromide, and visualized directly under ultraviolet illumination.

### LOH assay

For LOH analysis, two polymorphic microsatellite markers (D3S1478, D3S1621) encompassing the chromosomal region 3p21 were evaluated in 50 tumor tissues and their matched normal salivary gland tissues using fluorescent dye-labeled specific primers (the primer sequences, annealing temperature and estimated product size for each primer pair are available in the National Center for Biotechnology Information UniSTS database). The amplified PCR products for multiple loci were detected with an ABI 3730 Genetic Analyzer (Applied Biosystems) and analyzed with GeneMapper software (version 4.0) and Peak Scanner Software (version 1.0). A given informative marker was considered to display LOH when a ≥1.5-fold difference was detected in the relative allele height ratio between the tumor and the normal tissues. LOH was considered to have occurred in tumors with locus loss both upstream and downstream of the *RASSF1A* gene.

### Mutation analysis by PCR and direct sequencing

To determine the presence of mutation in tissues, all six exons of the *RASSF1A* gene were amplified using six primer pairs (sequences are listed in Table S1 of File S1). PCR was carried out in 25 µl reaction volume with TaKaRa Ex Taq (TaKaRa Biotechnology). For exons 2, 5 and 6, three-step PCR was used, and for exons 1, 3 and 4, touch-down PCR was adopted. All PCR products were subjected to direct sequencing using an ABI 3730XL Sequencer (Applied Biosystems).

### Follow-up information

The follow-up information of the 167 cases was collected from 2 to 132 months (mean 55 months). At the end of the follow-up, 40 patients (24%) had died (37 died of ACC and three died of other causes) and 127 patients (76%) were still alive. Sixty-six patients (40%) showed distant metastases (of lungs, liver, bone and brain) and 23 patients (14%) showed local recurrences of the disease.

### Statistical analysis

χ^2^ or Fisher exact tests were used to study the association between the categorical parameters and the clinicopathological parameters. Time-to-event analysis involved a log-rank testing of a Kaplan-Meier curve. The association between clinicopathological factors and patient outcome was estimated by a Cox proportional hazards regression model. The statistical analyses were performed with the software system of SPSS version 13.0. A 2-tailed *P*-value ≤0.05 was regarded as statistically significant.

## Supporting Information

File S1 **Table S1.** PCR primer sequences for bisulfite sequencing, MSP and mutation. **Table S2.** Clinicopathological details of 167 patients with ACC and promoter methylation, LOH of RASSF1A in tumor tissues.
(DOCX)
