# Supplementary material for: RASSF1A Promoter Hypermethylation Is a Strong Biomarker of Poor Survival in Patients with Salivary Adenoid Cystic Carcinoma in a Chinese Population
Source: PLoS One. 2014 Oct 10;9(10):e110159. doi: 10.1371/journal.pone.0110159 (PMC4193867; doi:10.1371/journal.pone.0110159)
Supplement: File S1 — Table S1. PCR primer sequences for bisulfite sequencing, MSP and mutation. Table S2. Clinicopathological details of 167 patients with ACC and promoter methylation, LOH of RASSF1A in tumor tissues. (DOCX) [file pone.0110159.s001.docx]

| Table S1. PCR primer sequences for bisulfite sequencing, MSP and mutation | | |
| --- | --- | --- |
| **Primer** | **Sequence(5'-3')** | **Product size** |
| Bisulfite sequencing |  |  |
| RASSF1A-BF | AGTTTTTGTATTTAGGTTTTTATTG | 191 |
| RASSF1A-BR | AACTCAATAAACTCAAACTCCCC |  |
| Methylation-specific PCR |  |  |
| RASSF1A-MF | AGTTTAGGTTTTTTCGATATGGTTC | 177 |
| RASSF1A-MR | CTACACCCAAATTTCCATTACG |  |
| RASSF1A-UF | TAGGTTTTTTTGATATGGTTTGG | 173 |
| RASSF1A-UR | CTACACCCAAATTTCCATTACAC |  |
| Mutation |  |  |
| Exon 1 |  |  |
| RASSF1A-1F | GCTTGCTAGCGCCCAAAG | 480 |
| RASSF1A-1R | TACGAGTGGAGTGCGACAAG |  |
| Exon 2 |  |  |
| RASSF1A-2F | ACATTAGAGTCCGCGTAGCAGT | 304 |
| RASSF1A-2R | ATCCTCGCCCTTCCCATAC |  |
| Exon 3 |  |  |
| RASSF1A-3F | GCCAAAGGCAGTCAGTTTCC | 373 |
| RASSF1A-3R | TGATAATAGGTTCCAGGTGAGATG |  |
| Exon 4 |  |  |
| RASSF1A-4F | CAGCCTAGCCCCAAGTAGAC | 499 |
| RASSF1A-4R | CAACAGCTTCCGCAAGTACA |  |
| Exon 5 |  |  |
| RASSF1A-5F | CCACCCTGCCCTATGTGA | 290 |
| RASSF1A-5R | CCTCCTCCAAGCCTTACTG |  |
| Exon 6 |  |  |
| RASSF1A-6F | AGCCCAGTCCTAGCTTGTG | 291 |
| RASSF1A-6R | CCTGCTGTCTGCCTTCCA |  |

| Table S2. Clinicopathological details of 167 patients with ACC and promoter methylation, LOH of RASSF1A in tumor tissues. | | | | | | | | | |
| --- | --- | --- | --- | --- | --- | --- | --- | --- | --- |
| **Case No.** | **Sex** | **Age (years)** | **Location** | **Grade/TNM Stage** | **Neural/Vascular Invasion** | **Therapy** | **Follow-up(mo)** | **RASSF1A promoter methylation** | **RASSF1A LOH** |
| 1 | F | 51 | sinus maxillaris | 1/III | Yes/Yes | O+R | NED(50) | U | No |
|  |  |  |  |  |  |  |  |  |  |
| 2 | F | 40 | sublingual | 2/I | Yes/Yes | O | NED(62) | U | No |
|  |  |  |  |  |  |  |  |  |  |
| 3 | F | 75 | floor of mouth | 2/III | Yes/No | O+R+C | Lung Met(4),AWD(27) | U | No |
|  |  |  |  |  |  |  |  |  |  |
| 4 | F | 50 | sublingual | 3/IV | Yes/No | O | Lung and bone Met(2),LR(2),DOD(10) | M | No |
|  |  |  |  |  |  |  |  |  |  |
| 5 | M | 30 | parotid | 2/III | Yes/Yes | O+R | NED(64) | M | No |
|  |  |  |  |  |  |  |  |  |  |
| 6 | F | 66 | palate | 2/II | Yes/No | O+R | Lung Met(14),LR(14),AWD(44) | U | No |
|  |  |  |  |  |  |  |  |  |  |
| 7 | F | 40 | parotid | 2/II | Yes/No | O+R | Lung Met(49), LR(58),AWD(58) | U | No |
|  |  |  |  |  |  |  |  |  |  |
| 8 | F | 67 | parotid | 1/II | No/No | O | NED(42) | U | No |
|  |  |  |  |  |  |  |  |  |  |
| 9 | M | 34 | submandibular | 1/III | Yes/Yes | O+R+C | Liver Met(68),AWD(73) | M | No |
|  |  |  |  |  |  |  |  |  |  |
| 10 | F | 82 | sublingual | 2/II | Yes/Yes | O | Brain Met(22),AWD(65) | U | No |
|  |  |  |  |  |  |  |  |  |  |
| 11 | F | 83 | sublingual | 2/II | Yes/No | O | Lung Met(11), DOD(12) | U | No |
|  |  |  |  |  |  |  |  |  |  |
| 12 | F | 47 | palate | 2/III | No/No | O+R | Lung Met(46), LR(46),AWD(80) | M | No |
|  |  |  |  |  |  |  |  |  |  |
| 13 | M | 57 | tongue | 2/III | Yes/No | O | Lung Met(36),AWD(70) | U | No |
|  |  |  |  |  |  |  |  |  |  |
| 14 | F | 47 | tongue | 2/III | Yes/No | O+R | NED(24) | U | Yes |
|  |  |  |  |  |  |  |  |  |  |
| 15 | M | 30 | parotid | 2/III | Yes/Yes | O+R | NED(64) | U | No |
|  |  |  |  |  |  |  |  |  |  |
| 16 | F | 48 | tongue | 2/III | Yes/No | O+R | LR(6),DOD(6) | U | Yes |
|  |  |  |  |  |  |  |  |  |  |
| 17 | M | 49 | parotid | 2/II | No/No | O+R | Lung Met(54),AWD(72) | M | No |
|  |  |  |  |  |  |  |  |  |  |
| 18 | M | 40 | submandibular | 2/II | Yes/No | O | NED(48) | U | No |
|  |  |  |  |  |  |  |  |  |  |
| 19 | M | 58 | buccal | 1/II | Yes/No | O+R | NED(46) | U | No |
|  |  |  |  |  |  |  |  |  |  |
| 20 | M | 69 | floor of mouth | 1/III | Yes/Yes | O+C | Lung Met(13),DOD(37) | M | Yes |
|  |  |  |  |  |  |  |  |  |  |
| 21 | F | 48 | buccal | 2/I | Yes/Yes | O+R | NED(45) | U | No |
|  |  |  |  |  |  |  |  |  |  |
| 22 | M | 59 | jaw | 1/III | No/Yes | O+R | NED(6) | M | No |
|  |  |  |  |  |  |  |  |  |  |
| 23 | F | 65 | parotid | 1/I | Yes/No | O+R | NED(44) | U | Yes |
|  |  |  |  |  |  |  |  |  |  |
| 24 | F | 72 | tongue | 1/III | Yes/Yes | O | LR(8), DOD(29) | M | No |
|  |  |  |  |  |  |  |  |  |  |
| 25 | M | 53 | tongue | 1/II | Yes/Yes | O+R | NED(43) | U | No |
|  |  |  |  |  |  |  |  |  |  |
| 26 | M | 60 | floor of mouth | 1/II | Yes/Yes | O+R | NED(43) | U | No |
|  |  |  |  |  |  |  |  |  |  |
| 27 | M | 45 | tongue | 1/III | Yes/Yes | O | NED(36) | U | No |
|  |  |  |  |  |  |  |  |  |  |
| 28 | F | 35 | sublingual | 1/II | Yes/Yes | O+R | NED(43) | U | No |
|  |  |  |  |  |  |  |  |  |  |
| 29 | F | 62 | jaw | 3/IV | No/Yes | O+R | Lung Met(2), AWD(2) | M | No |
|  |  |  |  |  |  |  |  |  |  |
| 30 | F | 68 | retro-molar | 3/IV | Yes/Yes | O+R | Lung Met(15),DOD(20) | M | Yes |
|  |  |  |  |  |  |  |  |  |  |
| 31 | M | 50 | floor of mouth | 2/II | Yes/No | O+R+C | Lung Met(24). AWD(40) | M | Yes |
|  |  |  |  |  |  |  |  |  |  |
| 32 | F | 72 | parotid | 3/III | Yes/Yes | O | NED(40) | M | No |
|  |  |  |  |  |  |  |  |  |  |
| 33 | M | 53 | tongue | 2/II | Yes/Yes | O+R | NED(41) | U | No |
|  |  |  |  |  |  |  |  |  |  |
| 34 | F | 49 | floor of mouth | 1/III | No/No | O+R | NED(2) | U | No |
|  |  |  |  |  |  |  |  |  |  |
| 35 | F | 59 | floor of mouth | 3/IV | Yes/No | O+C | Lung Met(1), AWD(57) | M | Yes |
|  |  |  |  |  |  |  |  |  |  |
| 36 | M | 45 | tongue | 2/II | Yes/Yes | O | NED(37) | U | No |
|  |  |  |  |  |  |  |  |  |  |
| 37 | M | 45 | tongue | 1/I | Yes/No | O+R+C | NED(34) | U | No |
|  |  |  |  |  |  |  |  |  |  |
| 38 | M | 66 | sublingual | 2/III | Yes/No | O+R | Lung Met(6),AWD(6) | M | No |
|  |  |  |  |  |  |  |  |  |  |
| 39 | M | 56 | palate | 2/IV | No/Yes | O+R | Liver Met(24),AWD(32) | U | Yes |
|  |  |  |  |  |  |  |  |  |  |
| 40 | F | 47 | buccal | 3/II | Yes/No | O+R | NED(30) | M | No |
|  |  |  |  |  |  |  |  |  |  |
| 41 | F | 58 | submandibular | 2/I | No/Yes | O+R | NED(30) | U | No |
|  |  |  |  |  |  |  |  |  |  |
| 42 | M | 38 | sinus maxillaris | 1/II | Yes/No | O+R+C | NED(30) | U | No |
|  |  |  |  |  |  |  |  |  |  |
| 43 | F | 62 | tongue | 2/II | Yes/No | O+R | Liver Met(29),AWD(31) | U | No |
|  |  |  |  |  |  |  |  |  |  |
| 44 | M | 70 | floor of mouth | 1/I | Yes/No | O+R | Liver Met(2),AWD(32) | U | No |
|  |  |  |  |  |  |  |  |  |  |
| 45 | M | 47 | parotid | 3/II | Yes/Yes | O+R | LR(13),Bone, Brain Met(17),DOD(27) | M | Yes |
|  |  |  |  |  |  |  |  |  |  |
| 46 | F | 47 | parotid | 2/IV | Yes/No | O+R | NED(28) | U | No |
|  |  |  |  |  |  |  |  |  |  |
| 47 | M | 70 | palate | 2/III | Yes/No | O+R+C | NED(28) | M | No |
|  |  |  |  |  |  |  |  |  |  |
| 48 | M | 58 | parotid | 2/II | Yes/Yes | O | NED(28) | U | No |
|  |  |  |  |  |  |  |  |  |  |
| 49 | M | 57 | tongue | 2/I | Yes/Yes | O+R | Lung Met(22), AWD(27) | U | No |
|  |  |  |  |  |  |  |  |  |  |
| 50 | M | 54 | floor of mouth | 2/IV | Yes/Yes | O+C | Lung Met(1),LR(16),AWD(27) | U | No |
|  |  |  |  |  |  |  |  |  |  |
| 51 | F | 31 | tongue | 2/I | Yes/No | O+R | NED(132) | U | / |
|  |  |  |  |  |  |  |  |  |  |
| 52 | F | 37 | buccal | 3/II | No/No | O+R | LR(68),DOD(72) | U | / |
|  |  |  |  |  |  |  |  |  |  |
| 53 | M | 67 | palate | 2/II | Yes/No | O+R | Lung Met(48),DOD(86) | M | / |
|  |  |  |  |  |  |  |  |  |  |
| 54 | F | 33 | retro-molar | 1/II | Yes/Yes | O+R | NED(130) | U | / |
|  |  |  |  |  |  |  |  |  |  |
| 55 | F | 29 | submandibular | 1/II | Yes/No | O | NED(128) | U | / |
|  |  |  |  |  |  |  |  |  |  |
| 56 | F | 70 | submandibular | 2/III | Yes/Yes | O+R | DOOD(104) | M | / |
|  |  |  |  |  |  |  |  |  |  |
| 57 | M | 49 | tongue | 2/III | Yes/Yes | O+R+C | NED(123) | U | / |
|  |  |  |  |  |  |  |  |  |  |
| 58 | M | 42 | palate | 3/IV | Yes/Yes | O+R | Lung Met(60),DOD(104) | M | / |
|  |  |  |  |  |  |  |  |  |  |
| 59 | F | 49 | palate | 2/II | Yes/No | O+R | LR(99),AWD(116) | U | / |
|  |  |  |  |  |  |  |  |  |  |
| 60 | M | 78 | palate | 2/II | No/No | O+R | Lung Met(60),DOD(68) | U | / |
|  |  |  |  |  |  |  |  |  |  |
| 61 | F | 45 | parotid | 3/III | Yes/No | O+R | NED(124) | U | / |
|  |  |  |  |  |  |  |  |  |  |
| 62 | F | 27 | parotid | 2/I | No/No | O+R | NED(119) | U | / |
|  |  |  |  |  |  |  |  |  |  |
| 63 | F | 30 | tongue | 1/II | Yes/No | O+R | Lung Met(60),AWD(120) | U | / |
|  |  |  |  |  |  |  |  |  |  |
| 64 | F | 68 | floor of mouth | 3/IV | Yes/Yes | O+R | Lung Met(60),AWD(120) | U | / |
|  |  |  |  |  |  |  |  |  |  |
| 65 | F | 65 | sublingual | 2/II | Yes/No | O+R | NED(118) | U | / |
|  |  |  |  |  |  |  |  |  |  |
| 66 | M | 40 | buccal | 2/IV | Yes/Yes | O+R | Lung Met(26),DOD(56) | U | / |
|  |  |  |  |  |  |  |  |  |  |
| 67 | M | 58 | sinus maxillaris | 2/III | No/No | O+R | NED(116) | U | / |
|  |  |  |  |  |  |  |  |  |  |
| 68 | M | 30 | sublingual | 1/II | Yes/No | O+R | NED(36) | M | / |
|  |  |  |  |  |  |  |  |  |  |
| 69 | M | 47 | submandibular | 1/II | Yes/Yes | O+R | NED(44) | U | / |
|  |  |  |  |  |  |  |  |  |  |
| 70 | M | 33 | tongue | 2/III | Yes/No | O+R | NED(102) | M | / |
|  |  |  |  |  |  |  |  |  |  |
| 71 | M | 40 | palate | 3/I | Yes/Yes | O+R | LN Met(85), NED(109) | U | / |
|  |  |  |  |  |  |  |  |  |  |
| 72 | F | 57 | tongue | 2/IV | Yes/No | O+R | Lung Met(60), DOD(89) | U | / |
|  |  |  |  |  |  |  |  |  |  |
| 73 | M | 58 | floor of mouth | 3/III | No/No | O+R | NED(106) | U | / |
|  |  |  |  |  |  |  |  |  |  |
| 74 | F | 46 | sublingual | 1/III | No/Yes | O+R | NED(103) | U | / |
|  |  |  |  |  |  |  |  |  |  |
| 75 | F | 45 | palate | 1/II | Yes/Yes | O+R | Lung Met(50),AWD(100) | U | / |
|  |  |  |  |  |  |  |  |  |  |
| 76 | M | 66 | palate | 3/II | Yes/Yes | O+R | Lung Met(13),DOD(68) | U | / |
|  |  |  |  |  |  |  |  |  |  |
| 77 | M | 40 | palate | 2/II | Yes/Yes | O+R | Lung Met(24),DOD(51) | U | / |
|  |  |  |  |  |  |  |  |  |  |
| 78 | F | 72 | submandibular | 3/II | Yes/No | O+R | LR(20), DOD(22) | M | / |
|  |  |  |  |  |  |  |  |  |  |
| 79 | M | 54 | parotid | 1/I | No/No | O+R | AWD(87) | M | / |
|  |  |  |  |  |  |  |  |  |  |
| 80 | M | 52 | sinus maxillaris | 1/II | No/No | O | Bone Met(32),DOD(41) | U | / |
|  |  |  |  |  |  |  |  |  |  |
| 81 | M | 54 | tongue | 1/III | Yes/No | O+R+C | NED(97) | U | / |
|  |  |  |  |  |  |  |  |  |  |
| 82 | F | 76 | floor of mouth | 1/II | Yes/No | O | NED(84) | M | / |
|  |  |  |  |  |  |  |  |  |  |
| 83 | F | 79 | sublingual | 1/II | Yes/No | O | NED(90) | U | / |
|  |  |  |  |  |  |  |  |  |  |
| 84 | F | 73 | palate | 2/I | No/Yes | O+C | LR(76),NED(82) | U | / |
|  |  |  |  |  |  |  |  |  |  |
| 85 | F | 43 | sublingual | 3/II | Yes/No | O+R+C | LR(22),Lung,Bone Met(22),AWD(80) | U | / |
|  |  |  |  |  |  |  |  |  |  |
| 86 | M | 49 | palate | 2/II | Yes/Yes | O | LR(16),NED(77) | M | / |
|  |  |  |  |  |  |  |  |  |  |
| 87 | M | 68 | sublingual | 2/IV | Yes/No | O+R+C | Brain Met(58),DOD(68) | M | / |
|  |  |  |  |  |  |  |  |  |  |
| 88 | M | 58 | floor of mouth | 2/I | Yes/No | O+R | NED(76) | M | / |
|  |  |  |  |  |  |  |  |  |  |
| 89 | F | 48 | parotid | 2/I | Yes/No | O+R | Lung Met(40),DOD(81) | M | / |
|  |  |  |  |  |  |  |  |  |  |
| 90 | F | 72 | palate | 2/II | Yes/No | O | NED(33) | U | / |
|  |  |  |  |  |  |  |  |  |  |
| 91 | F | 36 | tongue | 1/I | No/No | O+R+C | NED(82) | U | / |
|  |  |  |  |  |  |  |  |  |  |
| 92 | M | 34 | submandibular | 1/III | Yes/Yes | O+R+C | Liver Met(68),AWD(73) | M | / |
|  |  |  |  |  |  |  |  |  |  |
| 93 | F | 40 | floor of mouth | 2/IV | Yes/Yes | O+R | LR(14),Lung Met(17),DOD(38) | M | / |
|  |  |  |  |  |  |  |  |  |  |
| 94 | F | 33 | palate | 2/II | Yes/Yes | O+R | DOOD(74) | U | / |
|  |  |  |  |  |  |  |  |  |  |
| 95 | M | 64 | buccal | 2/II | Yes/No | O | Lung Met(44),DOD(49) | U | / |
|  |  |  |  |  |  |  |  |  |  |
| 96 | F | 61 | jaw | 3/III | Yes/Yes | O+R | LR(24),Brain(24),DOD(48) | U | / |
|  |  |  |  |  |  |  |  |  |  |
| 97 | M | 43 | tongue | 1/II | No/No | O+R | NED(77) | U | / |
|  |  |  |  |  |  |  |  |  |  |
| 98 | M | 63 | tongue | 2/II | No/No | O+R | Lung Met(46),DOD(70) | U | / |
|  |  |  |  |  |  |  |  |  |  |
| 99 | F | 40 | sublingual | 1/II | Yes/No | O+R | NED(70) | U | / |
|  |  |  |  |  |  |  |  |  |  |
| 100 | M | 37 | buccal | 2/III | Yes/No | O+R+C | NED(75) | M | / |
|  |  |  |  |  |  |  |  |  |  |
| 101 | M | 64 | tongue | 3/II | Yes/Yes | O+R | Lung Met(12),DOD(28) | M | / |
|  |  |  |  |  |  |  |  |  |  |
| 102 | M | 82 | sublingual | 2/II | Yes/Yes | O | Brain Met(22),AWD(65) | U | / |
|  |  |  |  |  |  |  |  |  |  |
| 103 | M | 35 | submandibular | 1/II | Yes/No | O+R | NED(72) | U | / |
|  |  |  |  |  |  |  |  |  |  |
| 104 | M | 66 | parotid | 1/III | Yes/No | O+R | NED(60) | U | / |
|  |  |  |  |  |  |  |  |  |  |
| 105 | F | 33 | sublingual | 2/I | Yes/No | O+R | Lung Met(23),AWD(45) | U | / |
|  |  |  |  |  |  |  |  |  |  |
| 106 | F | 57 | sublingual | 3/II | No/Yes | O+R | Lung Met(19),DOD(27) | M | / |
|  |  |  |  |  |  |  |  |  |  |
| 107 | M | 66 | tongue | 1/III | Yes/No | O+R | Lung Met(24),DOD(46) | U | / |
|  |  |  |  |  |  |  |  |  |  |
| 108 | M | 53 | tongue | 1/I | No/No | O+C | NED(58) | U | / |
|  |  |  |  |  |  |  |  |  |  |
| 109 | M | 57 | tongue | 2/III | No/No | O+R+C | Lung Met(7),AWD(46) | U | / |
|  |  |  |  |  |  |  |  |  |  |
| 110 | F | 42 | retro-molar | 2/I | Yes/No | O | NED(58) | U | / |
|  |  |  |  |  |  |  |  |  |  |
| 111 | F | 43 | sublingual | 1/III | Yes/No | O+R | NED(58) | U | / |
|  |  |  |  |  |  |  |  |  |  |
| 112 | M | 72 | floor of mouth | 2/II | No/No | O+R | DOOD(7) | U | / |
|  |  |  |  |  |  |  |  |  |  |
| 113 | F | 43 | submandibular | 2/II | Yes/No | O+R | NED(65) | U | / |
|  |  |  |  |  |  |  |  |  |  |
| 114 | M | 48 | floor of mouth | 1/II | Yes/Yes | O+R+C | NED(56) | U | / |
|  |  |  |  |  |  |  |  |  |  |
| 115 | M | 53 | parotid | 1/II | Yes/Yes | O+R+C | Lung,Liver Met(36),AWD(70) | M | / |
|  |  |  |  |  |  |  |  |  |  |
| 116 | F | 62 | palate | 3/I | Yes/Yes | O+R | NED(55) | U | / |
|  |  |  |  |  |  |  |  |  |  |
| 117 | M | 35 | tongue | 2/III | Yes/Yes | O+R | Lung Met(4),AWD(65) | M | / |
|  |  |  |  |  |  |  |  |  |  |
| 118 | F | 59 | sinus maxillaris | 2/III | No/Yes | O+R+C | NED(64) | U | / |
|  |  |  |  |  |  |  |  |  |  |
| 119 | M | 30 | sinus maxillaris | 1/II | Yes/No | O+R+C | LR(36),NED(53) | U | / |
|  |  |  |  |  |  |  |  |  |  |
| 120 | M | 44 | floor of mouth | 1/II | Yes/No | O+C | Lung Met(13),AWD(60) | M | / |
|  |  |  |  |  |  |  |  |  |  |
| 121 | M | 70 | sublingual | 3/II | Yes/Yes | O | NED(48) | U | / |
|  |  |  |  |  |  |  |  |  |  |
| 122 | F | 57 | sublingual | 1/II | Yes/Yes | O+R | NED(10) | M | / |
|  |  |  |  |  |  |  |  |  |  |
| 123 | F | 46 | floor of mouth | 1/II | No/No | O+R | NED(57) | U | / |
|  |  |  |  |  |  |  |  |  |  |
| 124 | F | 61 | jaw | 1/II | Yes/No | O+R | NED(58) | U | / |
|  |  |  |  |  |  |  |  |  |  |
| 125 | F | 74 | jaw | 2/III | Yes/No | O | LR(26),NED(48) | U | / |
|  |  |  |  |  |  |  |  |  |  |
| 126 | M | 45 | palate | 2/I | Yes/No | O | LN Met(13),NED(52) | U | / |
|  |  |  |  |  |  |  |  |  |  |
| 127 | F | 41 | palate | 1/II | Yes/Yes | O | NED(51) | U | / |
|  |  |  |  |  |  |  |  |  |  |
| 128 | M | 46 | parotid | 1/III | Yes/Yes | O | NED(49) | U | / |
|  |  |  |  |  |  |  |  |  |  |
| 129 | M | 54 | sinus maxillaris | 2/III | Yes/No | O | Lung Met(48),AWD(48) | M | / |
|  |  |  |  |  |  |  |  |  |  |
| 130 | M | 55 | submandibular | 2/IV | No/No | O | Lung Met(1),DOD(12) | M | / |
|  |  |  |  |  |  |  |  |  |  |
| 131 | F | 57 | sublingual | 2/II | Yes/Yes | O | LR(55),NED(60) | M | / |
|  |  |  |  |  |  |  |  |  |  |
| 132 | M | 40 | palate | 1/II | Yes/No | O | NED(46) | M | / |
|  |  |  |  |  |  |  |  |  |  |
| 133 | M | 60 | sinus maxillaris | 3/III | No/Yes | O | LR(6),LN Met(6),DOD(12) | U | / |
|  |  |  |  |  |  |  |  |  |  |
| 134 | M | 49 | floor of mouth | 2/IV | Yes/No | O+R | Lung Met(48),AWD(48) | M | / |
|  |  |  |  |  |  |  |  |  |  |
| 135 | F | 52 | palate | 2/III | Yes/No | O+R | LR(6),LN,Bone Met(6),DOD(39) | U | / |
|  |  |  |  |  |  |  |  |  |  |
| 136 | M | 79 | tongue | 3/III | No/Yes | O+R | Lung Met(27), DOD(48) | U | / |
|  |  |  |  |  |  |  |  |  |  |
| 137 | M | 38 | sinus maxillaris | 3/III | Yes/Yes | O+R | Lung Met(24), DOD(46) | M | / |
|  |  |  |  |  |  |  |  |  |  |
| 138 | M | 68 | sublingual | 3/IV | Yes/Yes | O+R+C | Lung,Bone Met(10),DOD(14) | M | / |
|  |  |  |  |  |  |  |  |  |  |
| 139 | M | 43 | submandibular | 2/II | Yes/Yes | O+R | NED(51) | M | / |
|  |  |  |  |  |  |  |  |  |  |
| 140 | F | 32 | palate | 2/II | Yes/Yes | O | NED(51) | U | / |
|  |  |  |  |  |  |  |  |  |  |
| 141 | M | 56 | buccal | 3/III | No/Yes | O+R | Lung Met(24),DOD(46) | M | / |
|  |  |  |  |  |  |  |  |  |  |
| 142 | M | 57 | palate | 2/II | Yes/Yes | O | Lung Met(25),AWD(50) | U | / |
|  |  |  |  |  |  |  |  |  |  |
| 143 | F | 53 | palate | 2/II | Yes/No | O | NED(49) | M | / |
|  |  |  |  |  |  |  |  |  |  |
| 144 | M | 42 | buccal | 2/III | Yes/Yes | O+R | Lung Met(24),DOD(46) | M | / |
|  |  |  |  |  |  |  |  |  |  |
| 145 | F | 40 | retro-molar | 2/III | Yes/No | O+R | NED(49) | M | / |
|  |  |  |  |  |  |  |  |  |  |
| 146 | M | 72 | sublingual | 2/III | Yes/No | O+R | NED(49) | U | / |
|  |  |  |  |  |  |  |  |  |  |
| 147 | M | 45 | tongue | 2/IV | Yes/Yes | O | NED(48) | U | / |
|  |  |  |  |  |  |  |  |  |  |
| 148 | F | 42 | palate | 2/II | Yes/No | O+R+C | NED(48) | U | / |
|  |  |  |  |  |  |  |  |  |  |
| 149 | M | 60 | floor of mouth | 2/III | Yes/No | O+R | NED(48) | U | / |
|  |  |  |  |  |  |  |  |  |  |
| 150 | M | 45 | submandibular | 2/II | Yes/Yes | O+C | Lung Met(1),AWD(46) | U | / |
|  |  |  |  |  |  |  |  |  |  |
| 151 | M | 49 | buccal | 1/II | Yes/No | O+R+C | NED(46( | U | / |
|  |  |  |  |  |  |  |  |  |  |
| 152 | F | 59 | palate | 3/III | Yes/No | O+R+C | NED(45) | U | / |
|  |  |  |  |  |  |  |  |  |  |
| 153 | F | 68 | parotid | 1/IV | Yes/Yes | O+R+C | Lung Met(1),AWD(45) | U | / |
|  |  |  |  |  |  |  |  |  |  |
| 154 | M | 33 | jaw | 1/III | Yes/No | O | NED(45) | U | / |
|  |  |  |  |  |  |  |  |  |  |
| 155 | M | 50 | floor of mouth | 2/III | Yes/Yes | O | Lung Met(32),AWD(45) | M | / |
|  |  |  |  |  |  |  |  |  |  |
| 156 | M | 53 | floor of mouth | 2/II | Yes/Yes | O+R+C | LR(6),Lung Met(6),DOD(44) | M | / |
|  |  |  |  |  |  |  |  |  |  |
| 157 | M | 70 | buccal | 1/I | Yes/No | O+R | NED(35) | U | / |
|  |  |  |  |  |  |  |  |  |  |
| 158 | F | 61 | sinus maxillaris | 3/III | Yes/Yes | O | Lung Met(10), DOD(16) | M | / |
|  |  |  |  |  |  |  |  |  |  |
| 159 | M | 28 | floor of mouth | 1/III | Yes/No | O | NED(44) | U | / |
|  |  |  |  |  |  |  |  |  |  |
| 160 | F | 59 | palate | 3/II | No/No | O+R | NED(44) | M | / |
|  |  |  |  |  |  |  |  |  |  |
| 161 | F | 65 | palate | 3/III | Yes/Yes | O+R | NED(42) | M | / |
|  |  |  |  |  |  |  |  |  |  |
| 162 | F | 41 | palate | 2/II | Yes/Yes | O | NED(40) | U | / |
|  |  |  |  |  |  |  |  |  |  |
| 163 | M | 77 | tongue | 3/III | Yes/Yes | O | DOD(2) | M | / |
|  |  |  |  |  |  |  |  |  |  |
| 164 | M | 46 | lip | 3/I | Yes/Yes | O | NED(26) | M | / |
|  |  |  |  |  |  |  |  |  |  |
| 165 | M | 38 | external auditory canal | 2/II | Yes/No | O+R | Lung Met(34), | M | / |
|  |  |  |  |  |  |  |  |  |  |
| 166 | M | 29 | palate | 1/II | Yes/No | O+R | NED(40) | M | / |
|  |  |  |  |  |  |  |  |  |  |
| 167 | F | 47 | floor of mouth | 1/II | Yes/No | O | NED(40) | U | / |
| Abbreviations: M, male; F, female; O, opreation; R: radiotherapy; C: chemotherapy; LR: local recurrence; NED: no evidence of disease; AWD: alive with disease; DOD: died of disease; DOOD: died of other disease; Met: metastasis. | | | | | | | | | |
|  | | | | | | | | | |
|  |  |  |  |  |  |  |  |  |  |
